# Supplementary material for: Geographic population genetic structure and diversity of Sophora moorcroftiana based on genotyping-by-sequencing (GBS)
Source: PeerJ. 2020 Aug 6;8:e9609. doi: 10.7717/peerj.9609 (PMC7676378; doi:10.7717/peerj.9609)
Supplement: Supplemental Information 5 [file peerj-08-9609-s005.docx]

The parameters for denovo assembly as follows:

ustacks -f -o -i -M 6 -m 2 -p 16 --deleverage

cstacks -s -n 4 -p 32

sstacks -s -c -p 16 -o

gstacks -P -M -t 16 （）

populations -P -M -t 16 -O

The calculated parameters of Hw, Ho, He, Fst by Stacks are as follows:

--vcf --fasta_loci --hwe --fstats -k
