# Supplementary figures and images for: Geographic population genetic structure and diversity of Sophora moorcroftiana based on genotyping-by-sequencing (GBS)
Source: PeerJ. 2020 Aug 6;8:e9609. doi: 10.7717/peerj.9609 (PMC7676378; doi:10.7717/peerj.9609)

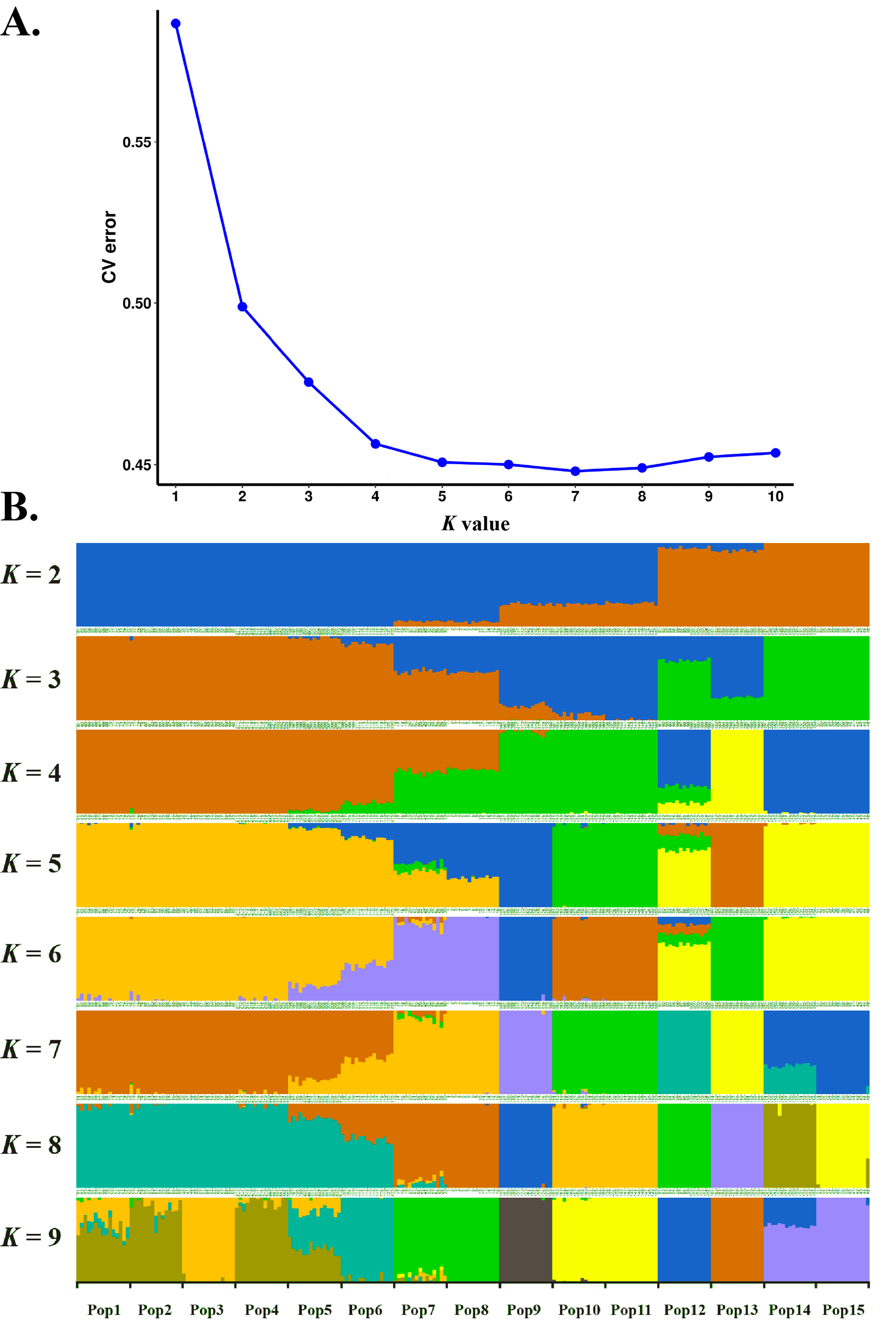

Supplement: Supplemental Information 6 — (A) The CV error varies among K values. (B) Clustering information for different individuals when K = 2 to 9. [file peerj-08-9609-s006.png]

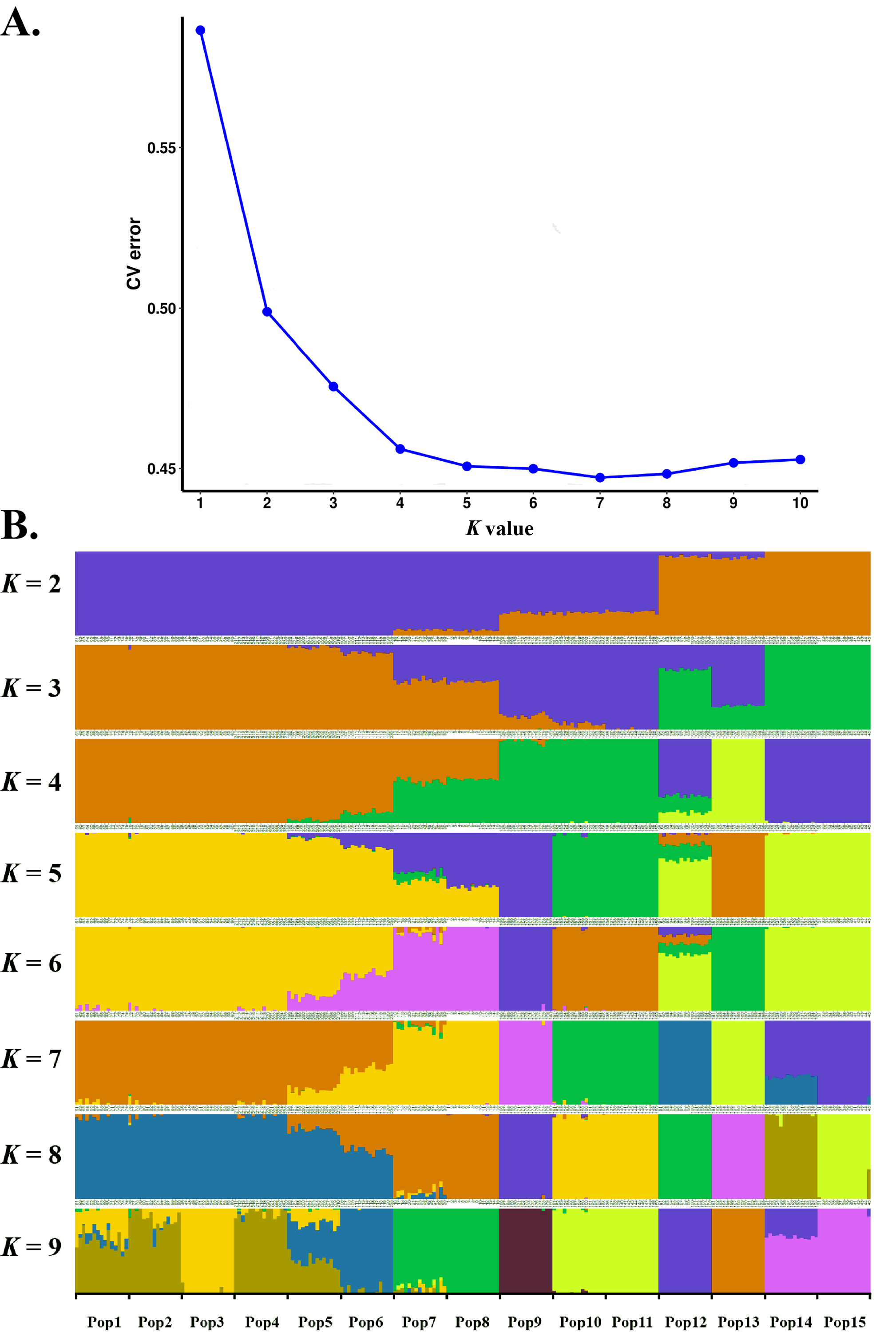

Supplement: Supplemental Information 7 — (A) The CV error varies among K values. (B) Clustering information for different individuals when K = 2 to 9. [file peerj-08-9609-s007.png]

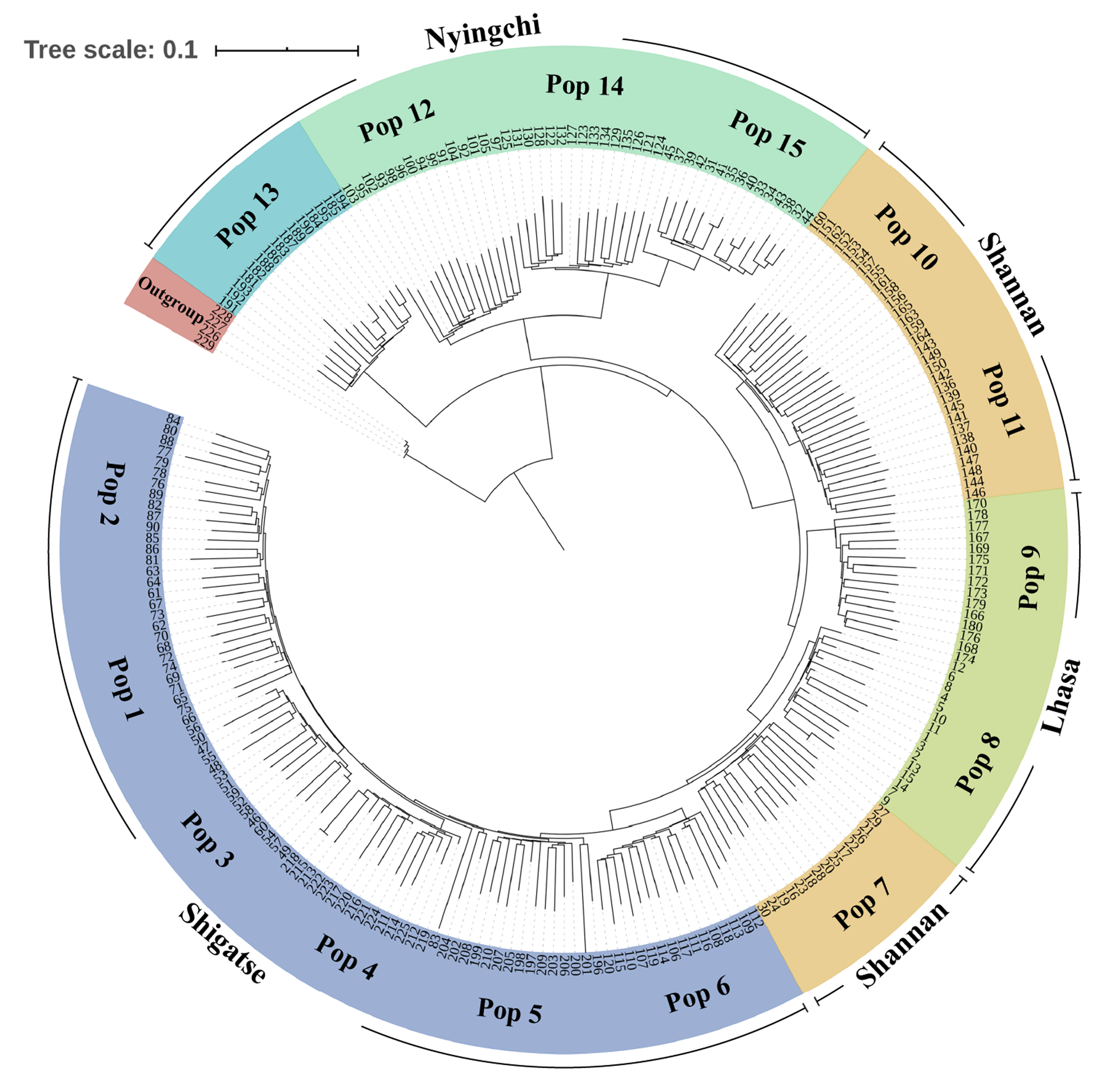

Supplement: Supplemental Information 8 — The tree was built based on the SNP data containing only the first SNP per locus. The periphery of the tree includes the population location information and the inner part indicates the individual number. Outgroup includes 4 S. davidii individuals, and the remaining populations are the S. moorcroftiana individuals. [file peerj-08-9609-s008.png]

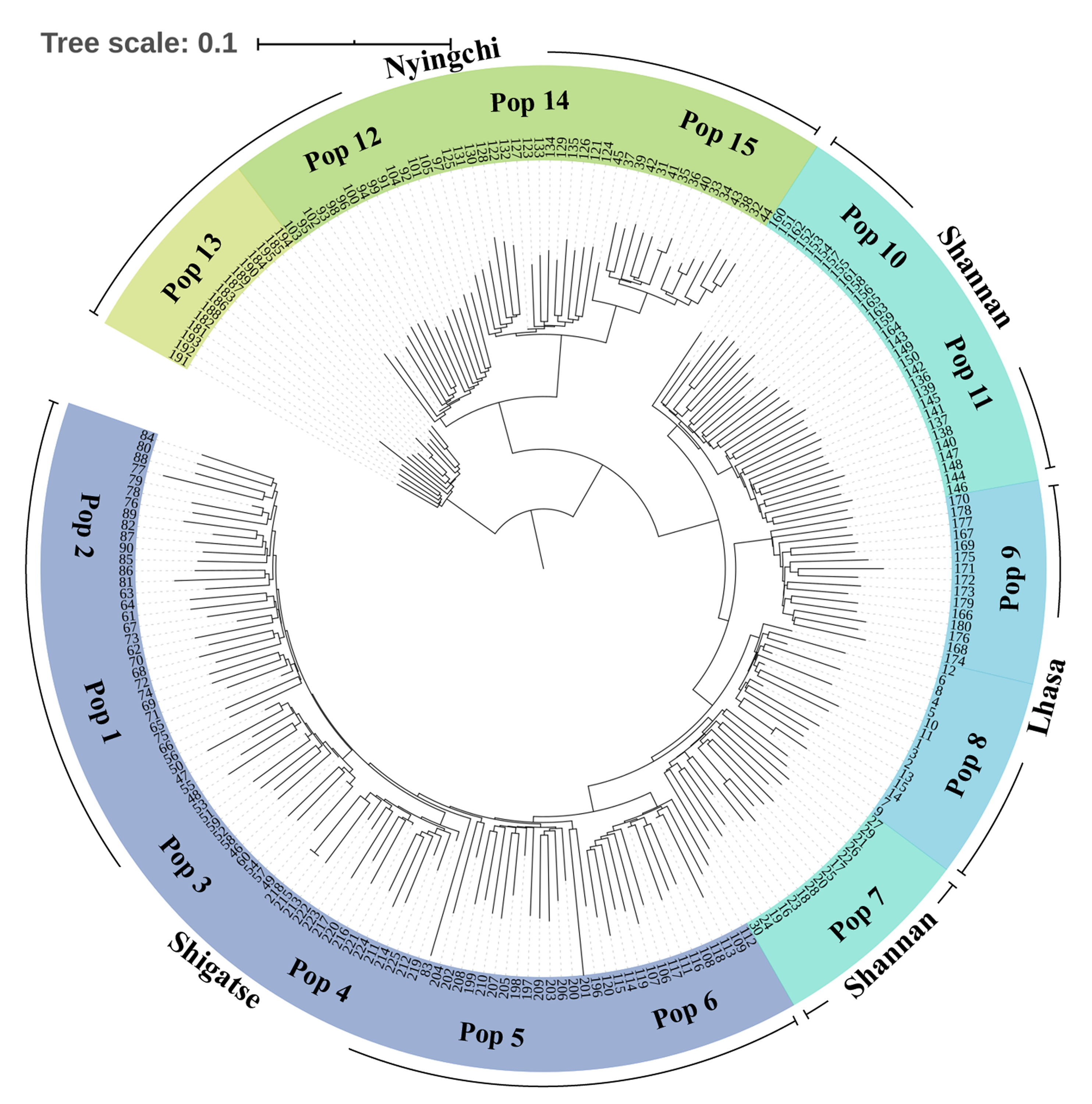

Supplement: Supplemental Information 9 — The tree was built based on the SNP data containing one random SNP per locus. The periphery of the tree includes the population location information and the inner part indicates the individual number. Outgroup includes 4 S. davidii individuals, and the remaining populations are the S. moorcroftiana individuals. [file peerj-08-9609-s009.png]

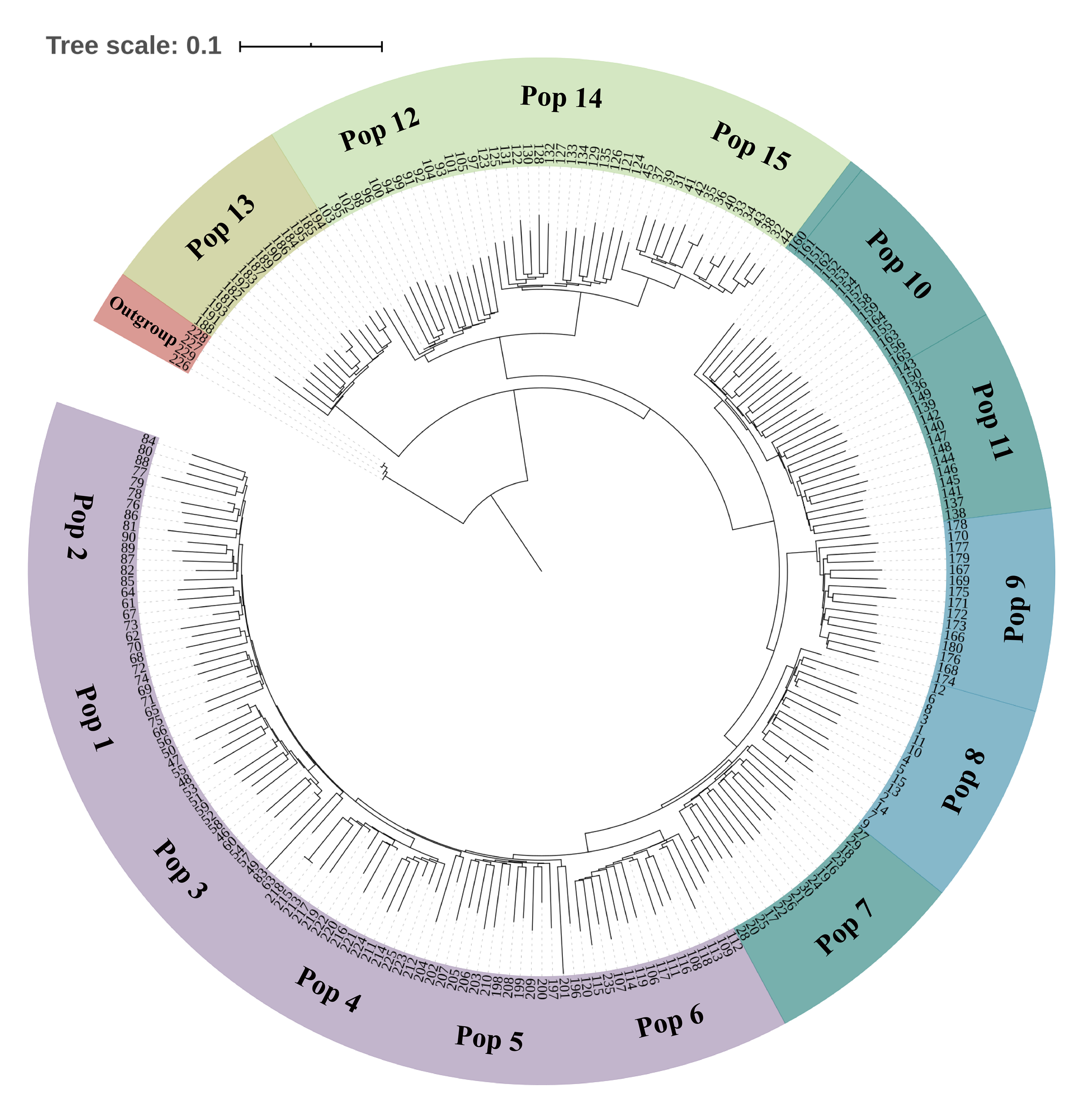

Supplement: Supplemental Information 10 — The tree was built based on the SNP data containing only the first SNP per locus. The periphery of the tree includes the 15 S. moorcroftiana populations’ location information, and the inner part indicates the 225 individuals’ number. [file peerj-08-9609-s010.png]

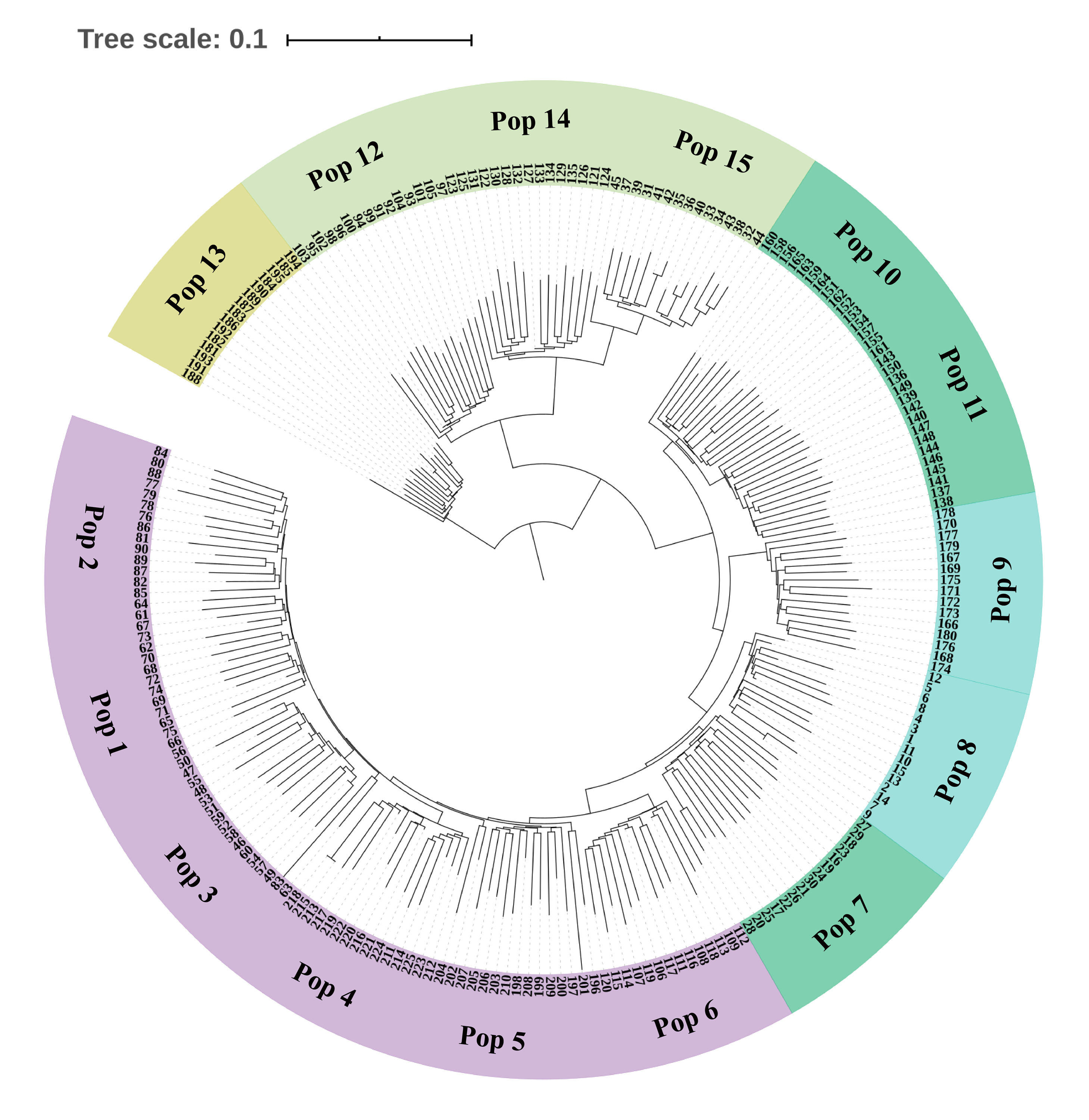

Supplement: Supplemental Information 11 — The tree was built based on the SNP data containing one random SNP per locus. The periphery of the tree includes the 15 S. moorcroftiana populations’ location information, and the inner part indicates the 225 individuals’ number. [file peerj-08-9609-s011.png]
